# Senescence Markers and Associated Transcriptomic Changes Are Expressed at Early Stages of Alzheimer’s Neuropathology but Are Not Independently Related to Dementia

**DOI:** 10.3390/ijms27156964

**Published:** 2026-08-03

**Authors:** Irina Vazquez-Villaseñor, Bridget Benson, Connor D. Richardson, Rachel Waller, Lydia M. Castelli, Julie E. Simpson, Fiona E. Matthews, Carol Brayne, Stephen B. Wharton

**Affiliations:** 1 Sheffield Institute for Translational Neuroscience, University of Sheffield, Sheffield S10 2HQ, UK; 2 Population Health Sciences Institute, Newcastle University, Newcastle upon Tyne NE1 7RU, UK; 3 Institute for Clinical and Applied Research, University of Hull, Hull HU6 7RX, UK; 4 Cambridge Public Health, School of Clinical Medicine, University of Cambridge, Cambridge CB2 0SP, UK

**Keywords:** senescence, p21, p16, ɣH2Ax, dementia, Alzheimer’s disease, transcriptomic analysis

## Abstract

Cellular senescence may affect the post-mitotic cells of the brain. We examined the expression of senescence markers, including p16, p21, γH2Ax and H3K9me3, in the frontal cortex of brain donations from the Cognitive Function and Ageing Study to assess their relationship to Alzheimer’s disease neuropathological change (ADNC) and dementia. p21, γH2Ax and H3K9me3 were expressed in pyramidal neurons and glia, whilst p16 was confined to glial cells. p21 and γH2Ax were correlated in neurons, and with p16 in glia. They did not increase with ADNC, tending to be higher at early Braak neurofibrillary tangle stages. Transcriptomic profiling of pyramidal neuron-enriched samples at low Braak stages showed that higher neuronal p21 expression was associated with altered pathways for neuronal function, neurodegeneration, protein homeostasis, mitochondrial dysfunction and synaptic signalling. In conclusion, the different expression profile of senescence markers in neurons and glia suggest possible differences in senescence-related mechanisms. Expression at lower ADNC stages suggests senescence may be important at earlier stages of Alzheimer’s pathogenesis, whilst transcriptomic changes suggest an impact on neuronal function. The lack of association of senescence markers with dementia status indicates that more work is needed to determine the value of senescence as a therapeutic target for dementia.

## 1. Introduction

Dementia in late life is associated with a variety of pathologies, of which Alzheimer’s disease neuropathological change (ADNC), Lewy body pathology and a range of vascular pathologies are the most common in a general population setting. Pathologies such as aging-related tau astrogliopathy (ARTAG), a form of tau pathology, and limbic-predominant age-related TDP-43 encephalopathy (LATE), a form of TDP-43 pathology are also commonly found at the oldest ages. Indeed, in the older general population, mixed pathology is a common substrate for dementia [1,2,3,4,5,6,7]. However, these disorders associated with classical pathological lesions do not fully explain dementia. For ADNC, there is increasing overlap in burdens of pathology in individuals with dementia compared to those without, whilst the brains of some individuals with dementia in life show low burdens of pathological lesions [8,9,10,11,12]. This suggests other factors may contribute to dementia.

Ageing is the most important risk factor for late-life dementia, the prevalence of which continues to rise with increasing age [13]. Cellular ageing itself results in processes that cause cell dysfunction [14], and these mechanisms could contribute to neuronal and glial dysfunction. Amongst these, cellular senescence is one of the key hallmarks of ageing. Senescence can be seen as a limited potential to divide in replicating cells (replicative senescence) but may also be induced by cell stress (stress-induced senescence) and has now been described in post-mitotic brain cells [15]. Senescence is associated with changes in cell morphology and in intracellular expression of molecules, including beta-galactosidase, DNA damage molecules and cell cycle regulatory proteins. A further hallmark of senescence is the secretion of a range of molecules, including inflammatory mediators, growth factors and matrix proteins, referred to as the senescence-associated secretory phenotype (SASP), which can drive tissue degeneration [16]. Expression of senescence-associated markers has been found in the human brain in ageing and neurodegeneration, including expression of beta-galactosidase, p16, p21 and p19 [17,18], and senescence markers have been found in association with ageing and with ADNC in mouse models [19,20]. Senescence is also a potential therapeutic target, with several approaches including senolytic drugs, enhancing immune clearance of senescence cells, modulating the SASP and genetic approaches (for experimental models) [16,21,22]. However, it remains unclear whether senescence could contribute to dementia either independently and/or as a component of the pathogenesis of neurodegenerative disorders.

We aimed to determine senescence in the ageing human brain, its relationship to ADNC and to dementia. We hypothesised that a neuronal senescence-like phenotype may be an independent contributor to dementia. We carried out this study using the well-characterised neuropathology cohort from the Cognitive Function and Ageing Study (CFAS). This is a population-representative study of those 65yrs and over, based in rural and urban centres in the UK. As the study is not based on cases preselected based on clinicopathological criteria, the use of this cohort design allows assessment of the variation of markers in the elderly brain and their relationship to burdens of pathology and dementia status (at death) using an unbiased approach [23].

We first examined the patterns of expression of a panel of putative senescence markers to determine how they relate to each other, and which may be the most appropriate to use to assess senescence in neurons and glia. Using selected markers, we then investigated how their expression changes with ADNC and how they are related to dementia status at death. We then used gene expression analysis to identify cellular pathway changes that are associated with higher expression of senescence markers.

## 2. Results

### 2.1. Expression and Comparison of Senescence Markers

**Figure 1:**
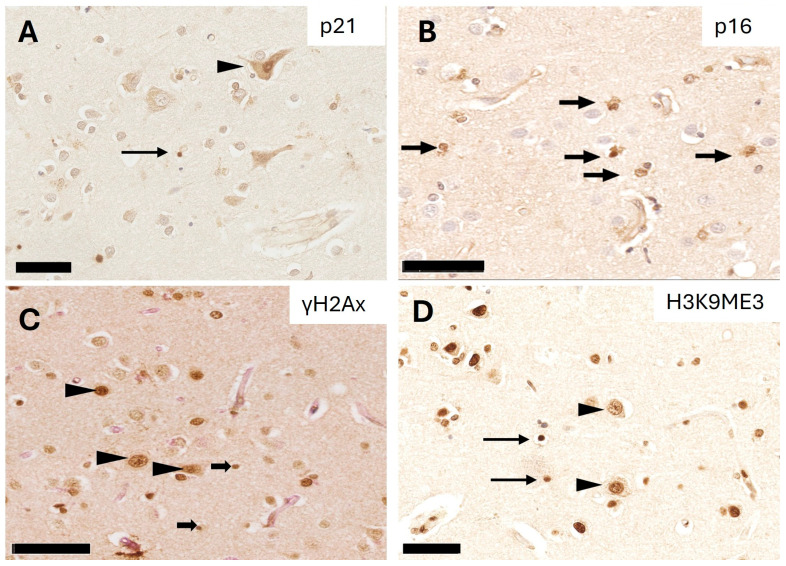
Immunohistochemistry for senescence markers. (**A**) p21 showing neuronal (arrowhead) and glial (arrows) staining. (**B**) p16 expressed in multiple glia (arrows). (**C**) γH2Ax expressed in neurons (arrowheads) and glia (arrows). (**D**) H3K9ME3 showing nuclear staining in neurons (arrowheads) and glia (arrows). Magnification bars 50 μm.

**Figure 2:**
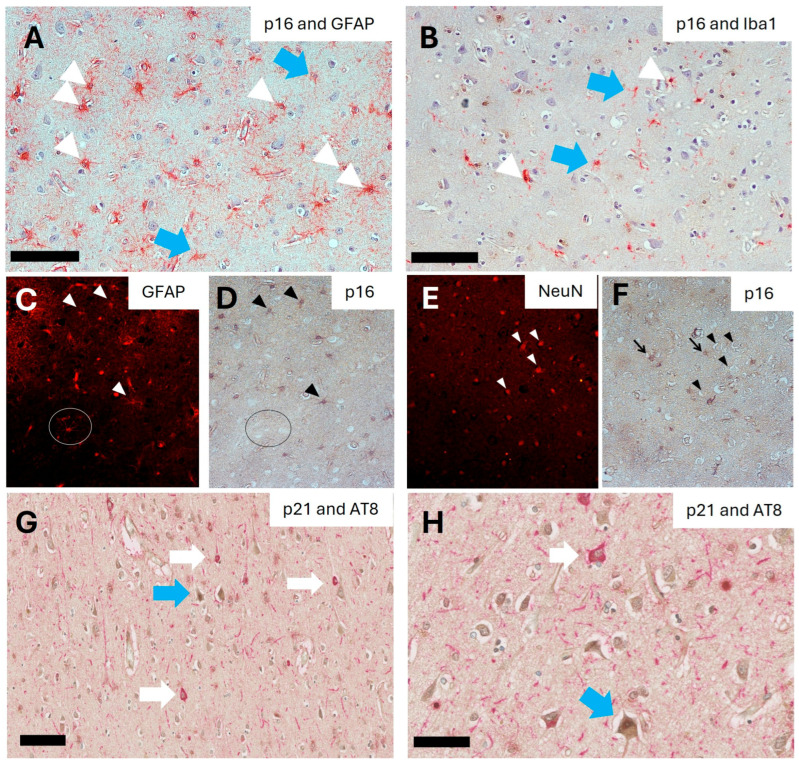
Immunohistochemistry double staining studies. (**A**) GFAP (red) and p16 (brown) showing GFAP+/p16+ (arrowhead) and GFAP+/p16- (blue arrow) glia. (**B**) Iba1 (red) and p16 (brown) showing Iba1+/p16+ (arrowhead) and Iba1+/p16- (blue arrow) microglia. (**C**–**F**) Single-channel images. (**C**,**D**) Staining for GFAP (immunofluorescence) and p16. GFAP positive astrocytes (white arrows) show corresponding labelling for p16 (black arrows), but there are GFAP positive astrocytes (circle) that do not label for p16. (**E**,**F**) Staining for neuronal marker NeuN (immunofluorescence, white arrowheads). These do not label for p16 (black arrowheads, (**F**)) but p16+ astrocytes (arrows) can be seen in the same field. (**G**,**H**) Dual detection of p21 and AT8. Blue arrows indicate p21 positive neuronal nuclei, while white arrows show AT8 positive neurons. AT8 did not colocalise with neurons positive for p21. Magnification bars (**A**,**B**,**G**) 100 μm, (**H**) 50 μm.

The markers (p16, p21, γH2Ax and H3K9me3) were initially assessed in the frontal cortex of the Cambridge subcohort (n = 99). p21 showed nuclear and cytoplasmic staining in neurons, small cells with morphology of glia, and small neurons. H3K9me3 and γH2Ax showed nuclear staining in neurons and glia. In contrast, p16 showed cytoplasmic and nuclear staining in glia only (Figure 1). Double staining for p16 with GFAP and Iba1 respectively showed co-localisation with some, but not all, GFAP-positive astrocytes, and also some co-localisation of p16 with microglia (Figure 2A–D). Sequential staining with NeuN confirmed that neurons did not label for p16 (Figure 2E,F).

**Figure 3:**
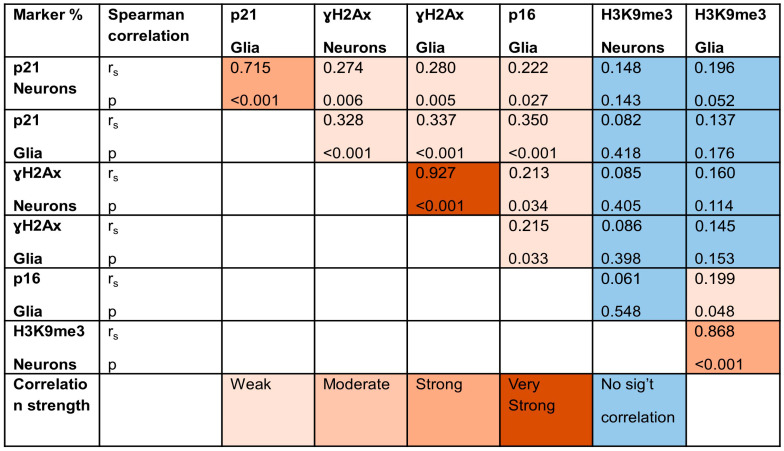
Correlation matrix showing correlation between different markers in neurons and glia. The bottom row shows the colour code for strength of associations.

We quantified each marker as % of cells positive, using size exclusion to distinguish pyramidal neurons and glia. All the markers in both glia and neurons showed skewed distributions within our population (Supplementary Figure S1). We assessed the relationships between markers using correlation analysis, hypothesising that, if these different measures were each detecting different aspects of the senescent phenotype, they would be correlated together (Figure 3). Neuronal and glial expression was correlated respectively for p21, γH2Ax and H3K9me3 (p16 being only glial), suggesting that both neurons and glia were affected similarly. There were also significant associations between p21, γH2Ax and p16 (for glia). H3K9me3 in neurons did not correlate with the other markers and showed only a very weak relation between its glial expression and p16. In view of the correlation results p21 and γH2Ax were chosen to be taken forwards to investigate the relationship to ADNC and dementia in our full cohort.

### 2.2. Variation of Senescence with Alzheimer’s Disease Neuropathological Change

Senescence markers (neuronal and glial p21 and γH2Ax) were available for 275 donation samples from the Cambridge, Nottingham and Newcastle centres where dementia status was known. There were 266 samples of neuronal and glial yH2Ax available where dementia status was known and 273 samples for p21 (Supplementary Table S1). For the analysis Braak staging (available in 219 samples), the Thal phase (available in 153 samples) was condensed into three categories and CAA (available in 151 samples) was grouped into tertiles.

**Figure 4:**
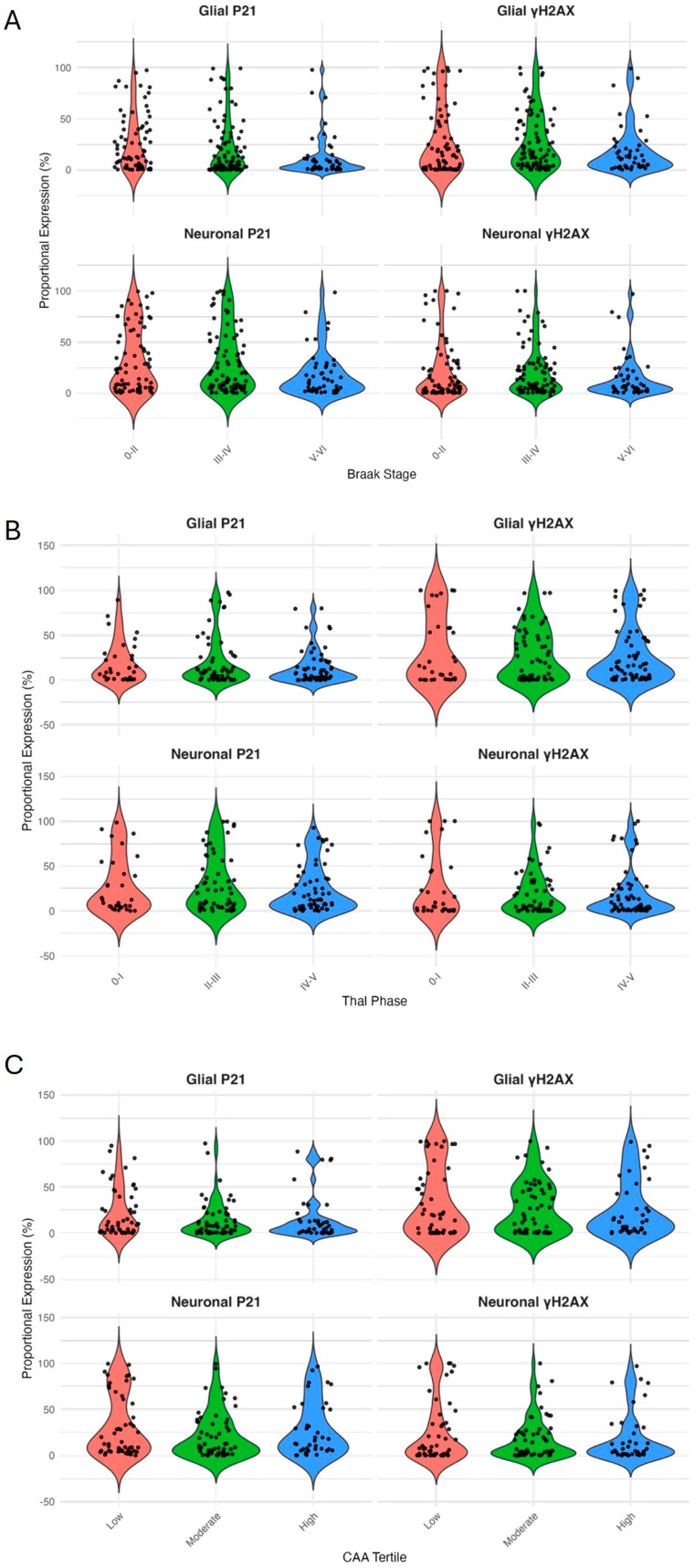
Violin plots showing the variation in glial and neuronal p21 and γH2Ax to ADNC stages. (**A**) Glial p21 (*p* = 0.02) and glial γH2Ax (*p* = 0.02) varied significantly with Braak NFT stages, whereas neuronal p21 (*p* = 0.08) and neuronal γH2Ax (*p* = 0.38) did not vary significantly. These markers in glial and neurons did not show significant variation with (**B**) Thal phase, or (**C**) CAA stage by tertile (see also Table 1 and Table 2).

Glial p21 was highest at the lowest Braak stages, decreasing with increasing Braak stage (*p* = 0.02). Glial γH2Ax was higher at low and intermediate stages, falling at the highest Braak stages (*p* = 0.02) (Table 1). Neuronal p21 and γH2Ax generally did not change across Braak stages, although neuronal p21 showed a borderline trend to decrease across Braak stages (*p* = 0.08) (Table 2). Neuronal and glial p21 showed the highest expression at the earliest Braak stages, declining progressively through higher stages. Neither p21 nor γH2Ax showed significant associations with the Thal phase or CAA (Figure 4). Double staining for p21 and AT8 did not show colocalisation of neuronal p21 with neurofibrillary tangles (Figure 2G,H).

### 2.3. Relationship of Senescence to Dementia Status

**Figure 5:**
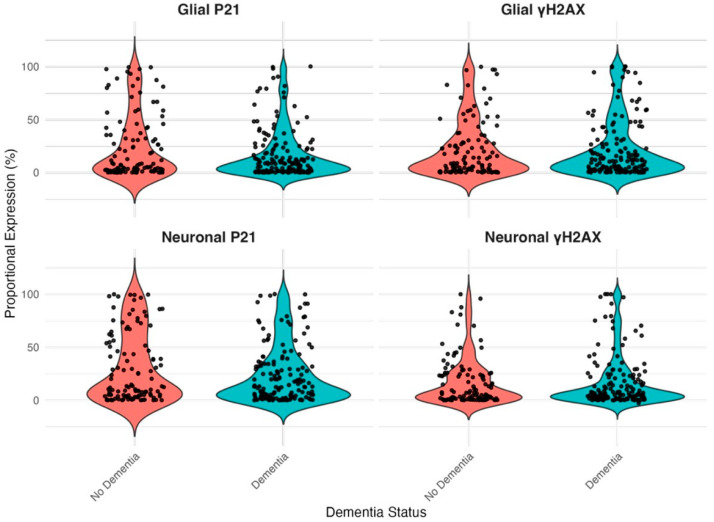
Violin plots showing variation in glial and neuronal p21 and γH2Ax between those with no dementia and with dementia at death. In an unadjusted logistic regression model with measures treated as continuous variables, none of the neuronal or glial markers (p21 or γH2Ax) were significantly associated with dementia risk. Models with senescence markers modelled as tertiles and adjusted for ADNC showed possible positive trends for moderate neuronal yH2Ax (OR = 2.58, 95% CI: 0.95–7.05) and moderate and high glial p21 and yH2Ax, but with wide confidence intervals crossing unity (see also Table 3).

There was no difference in expression of either p21 or γH2Ax in neurons or glia between those with and without dementia at death, and markers in both cell types showed wide variability (Figure 5). In both an unadjusted logistic regression model and a model adjusted for ADNC, none of the neuronal or glial markers (p21 or γH2Ax) were significantly associated with dementia risk (Table 3). Adjusted models showed possible positive trends for moderate neuronal yH2Ax (OR = 2.58, 95% CI: 0.95–7.05) and glial p21 and yH2Ax, but wide confidence intervals crossing unity indicated no robust evidence for an association.

### 2.4. Gene Expression Changes Associated with High Measures of Senescence

We now wish to determine whether higher levels of p21 are associated with alterations in pathways in neurons that may be relevant to neuronal dysfunction, and hence dementia and/or neurodegeneration. Given that neuronal senescence expression tended to be higher at earlier stages of ADNC progression, and to avoid the confounding effects of ADNC, we focused on low Braak stage cases (Braak NFT stages 1–2), comparing the transcriptomic profiles of cases with high vs. low p21 expression to further evaluate a potential role for this marker in pathways relevant to neurodegeneration and dementia.

**Figure 6:**
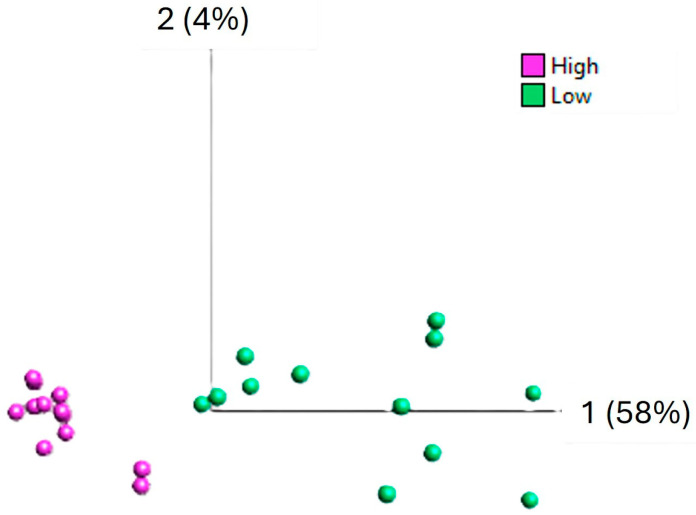
Principal Component Analysis (PCA) of neuronal transcripts showing separation of differentially expression genes between pyramidal and small neurons with high (purple) and low (green) measures of senescence.

Principal component analysis (PCA) of the differentially expressed genes (DEGs) revealed that cases with high expression of neuronal p21 clustered together and separated from cases with low levels of neuronal senescence (Figure 6). All samples passed the rigorous quality control procedures. The expression datasets are freely available at Gene Expression Omnibus, accession number (GSE310124).

A total of 3444 transcripts (3053 up-regulated and 391 down-regulated) were significantly differentially expressed in pyramidal and small neurons from cases with high levels of neuronal senescence. Pathway analysis identified significant dysregulation of transcripts associated with neurodegeneration, protein homeostasis, mitochondrial dysfunction, oxidative stress and synaptic signalling (Supplementary Table S2). Functional grouping analysis identified significant dysregulation of genes associated with several biological processes, including the electron transport chain and autophagy (Supplementary Table S3).

**Figure 7:**
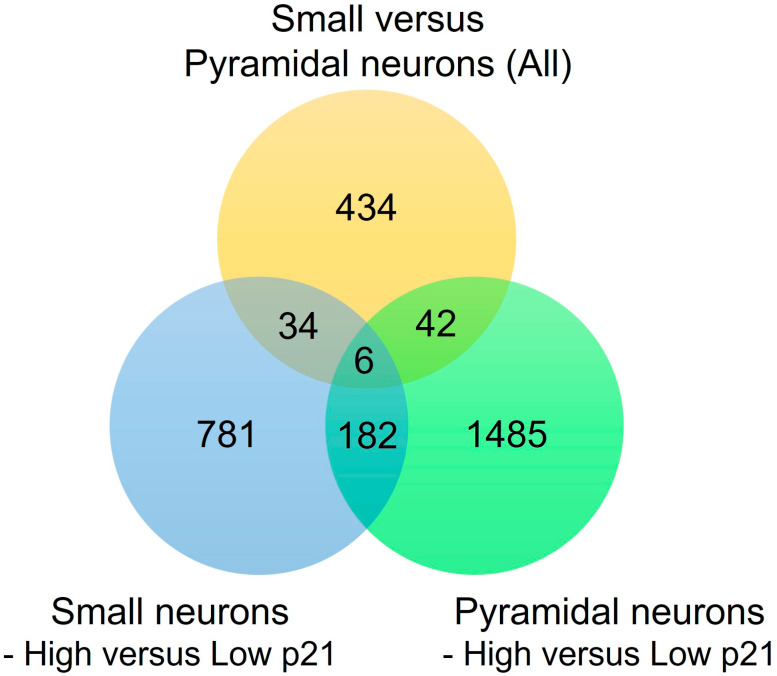
Venn diagrams showing gene expression comparisons of small vs. pyramidal neurons (irrespective of p21) and for high vs. low p21 in small neurons and in pyramidal neurons separately. In response to high levels of p21, 1003 genes were differentially expressed by small neurons and 1715 genes by pyramidal neurons, with an overlap of 188 DEGs. A total of 516 transcripts were differentially expressed in small versus pyramidal neurons, with six DEGs shared between these and the overlap between high vs. low p21 in both pyramidal and small neurons.

Further interrogation of the datasets, performed as a separate analysis regardless of p21 levels, identified 516 DEGs (207 up-regulated and 309 down-regulated) in small versus pyramidal neurons (Figure 7). KEGG pathway analysis and gene annotated clustering did not identify any significant dysregulated pathways or functional groups, where all Benjamini corrected *p*-values were >0.05. Only six DEGs were shared between these five hundred and sixteen DEGs and the overlap between high vs. low p21 in pyramidal neurons and high vs. low in small neurons, suggesting that the transcriptomic changes observed in the p21 analysis are due to p21 and not a cell-type-dependent approach.

The datasets were further investigated to determine the impact of senescence on pyramidal and small neurons separately (Figure 7). In pyramidal neurons, 1715 transcripts (1515 up-regulated and 200 down-regulated) were significantly differentially expressed in cases with high versus low levels of p21. Pathway analysis identified significant dysregulation of transcripts associated with neurodegeneration, mitochondrial dysfunction, oxidative stress and metabolic pathways (Supplementary Table S4). Functional annotation clustering identified significant dysregulation of genes associated with several biological processes, including the respiratory chain (Supplementary Table S5).

In small neurons, 1003 transcripts (733 up-regulated and 270 down-regulated) were significantly differentially expressed in cases with high versus low levels of p21. KEGG pathway analysis and gene annotated clustering did not identify any significant dysregulated pathways or functional groups, where all Benjamini corrected *p*-values were >0.05. However, 188 (11%) of the DEGs in pyramidal neurons were shared with the DEGs in the small neurons, 186 of which had the same directional change (167 up-regulated, 19 down-regulated), suggesting that there may be similar trends in both neuronal types.

## 3. Discussion

We here describe the distribution of putative markers of senescence in a population-representative cohort of the postmortem ageing human brain. H3K9me3, p21 and γH2Ax are expressed in both neurons and glia, with p16 restricted to glia. As in our previous study of motor neuron disease [18], we found p16 expression only in glia using this antibody, suggesting that mechanisms of senescence may differ between neurons and glia. p21 and γH2Ax (and p16 for glia) are highly correlated supporting the idea that they may be labelling different aspects of senescence in these cells, whereas H3K9me3 did not correlate well with other markers. In view of this, we used p21 and γH2Ax in the larger cohort. Given the variation between these markers, it would be interesting to carry out direct co-localisation studies in further work.

We show that, although there is considerable variability, these markers showed a trend to be more highly expressed at the lower stages of ADNC (as defined by Braak tangle stage but not by Thal Abeta phase or CAA stage) and that expression does not show an independent relationship to dementia. As markers were expressed more at early stages of ADNC, we hypothesised that senescence expression at this stage may be associated with changes that could contribute to AD progression and dementia. Using transcriptomic analysis, we show that high neuronal p21 expression significantly impacts pathways related to neurodegeneration, protein homeostasis, mitochondrial dysfunction and synaptic signalling. This was particularly related to changes in pyramidal neurons rather than small neurons.

Expression of these markers provides supportive evidence that a senescence-like mechanism can occur in post-mitotic cells in the brain. However, it is important to acknowledge that, whilst the expression of these markers could indicate senescence, they may also reflect cell stress, DNA damage or cell cycle changes as part of a DNA damage response independently [24,25]. Furthermore, senescence is a complex phenotype which may vary between cells, so a panel of makers is required [15,26,27]. The correlation between p21 and p16 (in glia), important drivers of senescence through p53 and Rb pathways respectively, and γH2Ax, a marker of DNA damage that is a key feature of senescence, suggests that this may be labelling a senescent-type phenotype in our cohort.

Senescence-associated heterochromatin foci (SAHF) are one of the most distinctive chromatin features of senescent cells in proliferating systems, characterised by punctate nuclear foci enriched in H3K9me3, macroH2A and HP1α which function to stably silence E2F target genes and enforce growth arrest [28,29]. In contrast, classical SAHF structures are rarely observed in post-mitotic neurons. Instead, neurons display more diffuse redistribution of heterochromatic marks, including H3K9me3, rather than discrete foci formation. These are associated with transcriptional repression, maintenance of neuronal identity, and stress responses [15,20,30] (although this transcriptional repression somewhat conflicts with our transcriptomic data, which detected more up-regulated than down-regulated transcripts in cases with high p21). Our finding that H3K9me3 does not correlate with p21 and γH2Ax, and only weakly with glial p16, suggests that in the ageing brain this marker reflects SAHF-like epigenetic remodelling processes or broader chromatin compaction, rather than canonical p16/Rb-driven senescence. This interpretation is consistent with reports of altered H3K9me3 distribution in Alzheimer’s disease, where it is linked to repression of neuronal and metabolic gene networks [31,32]. Taken together, these observations highlight that while H3K9me3 provides evidence of chromatin remodelling relevant to disease mechanisms, its pleiotropy limits its specificity as a senescence marker in the human brain.

We also tested senescence-associated β-galactosidase expression, which has been associated with senescence in the brain [20] and GL13 [33], which detects lipofuscin. However, we could not generate consistent and quantifiable staining with these and so did not test them further. Indeed, several studies have questioned senescence-associated β-galactosidase expression as a senescence marker in brain tissue, where it correlates poorly with other senescence markers [19,34]. Additionally, GL13 positivity appears to track chronic stress and can persist when DNA damage responses (e.g., after *H. pylori* infection) normalise, suggesting that it reports cumulative metabolic/lysosomal burden, rather than senescence per se [35].

In this study we demonstrate that these markers, expressed in both neurons and glia, do not accumulate into later stages of ADNC, and indeed are higher at early stages of pathology, suggesting that senescence mechanisms may be early in Alzheimer’s disease progression. Furthermore, there is higher inter-individual variability in these markers at lower ADNC stages, which decreases at higher stages. These findings are consistent with our previous findings with DNA damage markers [36]. Oxidative damage, an important driver of stress-related senescence, is an early feature of AD progression [37,38]. In a mouse tauopathy model, p16^INK4a^ expressing glial and microglial cells accumulated before the appearance of neurofibrillary tangles [22]. Our data, along with these studies, thus suggest that senescence (and oxidative damage) may be important earlier in the pathological progression. The apparent decline at later stages could also reflect selective neuronal loss or immune-mediated clearance of senescent cells.

Expression of the various markers in both neurons and glia in our study is at variance with some other studies. Some studies have found expression of senescence markers in neurons, including p21 and p16 in association with ADNC, and particularly correlating with neurofibrillary tangle formation in neurons [19,39]. Other studies have found that senescence markers are found particularly in glia, with little neuronal expression [17,21,22,40]. The restriction of p16 expression to glia and microglia in our study is consistent with p16 glial expression in many of these studies and may suggest a particular role for the p16/Rb pathway in glia rather than neurons. However, p16 expression was found to be predominantly neuronal in the 5XFAD Aβ-expressing mouse model, where its expression correlates inversely with cognitive measures [41]. Expression of p16 in endothelial cells and microglia has also been implicated in a murine tauopathy model [42]. The variability observed between studies is an issue in determining the relationship of senescence to ADNC development. This likely relates to variations in stages of disease included in studies, human tissue versus different animal/cell models and technical variations, for example in antibodies used. This may reflect the complexity of senescence, mechanisms of which may vary between cell types, diseases and models [26]. Currently, therefore, there is not a clear picture of the relationship of senescence to ADNC, although there are cell mechanisms that provide biological plausibility and there is model evidence that senolytics and genetic abrogation of senescence can improve pathological and cognitive measures [21,22], supporting the importance of senescence.

To seek evidence that senescence may be associated with disease-relevant mechanisms at early stages of ADNC, we compared the transcriptomes of neuronal-enriched samples between high and low p21 expressing cases in low Braak NFT stage cases. This identified the significant dysregulation of cellular pathways related to neurodegeneration, protein homeostasis, mitochondrial dysfunction, oxidative stress and synaptic signalling, suggesting that higher expression of senescence markers is associated with disease-relevant pathways. Mitochondrial dysfunction particularly is associated with expression of senescence markers in the brain in a number of studies [43,44,45], suggesting that this is an important common mechanistic pathway, along with alterations to DNA damage pathways, DNA/RNA synthesis, glucose and fatty acid metabolism and endoplasmic reticulum stress [19,21,40,46]. Thus, expression of senescence markers in the brain in our study is associated with alteration of senescent pathways and disease-relevant pathways. Interestingly, our study did not identify inflammation as an altered pathway, which is identified in other studies. It is possible that this relates to the early stage of the pathology with induction of inflammation being more downstream. The absence of enrichment for inflammatory pathways, despite their frequent association with senescence [27], suggests that the transcriptomic consequences of senescence in early ADNC may initially involve metabolic and mitochondrial processes, with inflammatory SASP responses emerging later or more prominently in glial populations.

As ageing is the most important risk factor for dementia, we also considered whether senescence per se might be independently related to dementia and therefore represent a therapeutic target. In our analyses, neuronal and glial senescence markers showed only weak associations with dementia status at death, with wide confidence intervals crossing unity. These findings indicate that, within this population-based cohort, we cannot conclude that senescence acts as an independent driver of dementia. However, the variability observed and the potential for modest effects mean that we cannot exclude a contributory role. It is possible that senescence acts primarily as an early-stage modifier of disease processes, interacting with classical ADNC rather than driving dementia directly. This interpretation is consistent with model studies in which senescent cell clearance or senolytic therapies ameliorate pathology and cognitive impairment [19,21]. Thus, while our data do not support senescence as an independent determinant of dementia risk, they reinforce its importance as an early mechanistic process and a potential therapeutic co-target alongside established AD pathologies.

Our study has several limitations. First, measurement of senescence in human brain tissue is challenging, and our approach relied primarily on immunohistochemistry. While molecules such as p16, p21 and γH2Ax are strongly associated with senescence, they are not specific and may participate in other cell processes distinct from senescence [27,46,47]. Second, we used cell size as a surrogate for glial vs. neuronal expression, as in our previous studies [36]. However, this does not allow certain identification of the subtypes of smaller cells and ideally quantification would be done on sections double-stained for glial, microglial and neuronal subtype markers. This would be an important avenue for further work but was beyond the scope of the current study with its large population-representative cohort. Third, as this is a postmortem, cross-sectional study, our results represent a single timepoint at death, and our assumption that different ADNC stages reflect a temporal continuum may not be valid for all individuals. Senescence phenotypes may fluctuate over time, and senescent cells could be selectively lost or cleared at later stages of disease. Fourth, our analyses were restricted to one brain region, so potential neuroanatomical variation in senescence expression remains unknown. Fifth, despite a large population-based sample, substantial inter-individual variability means our study may be underpowered to detect modest effects. Finally, whilst we identified senescence-like phenotypes in neurons, our transcriptomic analyses did not assess glial populations, which may display distinct senescence pathways, particularly via the p16/Rb axis. Future work should address glial senescence to provide a more complete picture of cell-type specific contributions.

Nonetheless, our findings contribute to the growing evidence that senescence-associated pathways are expressed in both neurons and glia in the ageing brain. Although we did not identify a clear independent relationship with dementia, the enrichment of senescence markers at early ADNC stages and their association with disease-relevant cellular pathways support a potential role in the initial phases of pathogenesis. These observations underscore the need for longitudinal and regionally comprehensive studies, as well as the development of in-life biomarkers of brain cell senescence, to clarify its temporal dynamics and evaluate its potential as an early therapeutic target.

## 4. Materials and Methods

### 4.1. Tissue Cohort

For the initial phase of comparison of a panel of senescence markers, formalin-fixed paraffin-embedded postmortem tissue from the frontal association cortex (BA8/9) was obtained from brains donated to one centre (Cambridge, n  =  99), of the CFAS neuropathology cohort. For the further phase to investigate the relation to dementia status, markers selected from the first phase were taken into brains from two additional centres (Nottingham and Newcastle).

Neuropathologists had previously examined the brains and categorised pathology as part of CFAS [23] based on several measures: Consortium to Establish a Registry for Alzheimer’s Disease (CERAD) stage [48] and Braak neurofibrillary tangle (NFT) stage [49]. Additional measures of Aβ pathology were also available on the cohort [50], including Thal Aβ phase [51]. Data on *APOE* status was also available [52]. National research ethics committee approval was granted for all procedures in this study (19/WM/0255) and approval was also given by the CFAS management committee.

### 4.2. Immunohistochemistry

Immunohistochemistry for senescence markers p16, p21 and H3K9me3, and for DNA damage marker γH2Ax was performed on formalin-fixed paraffin-embedded (FFPE) tissue using a standard avidin–biotin horseradish peroxidase enzyme complex method (Vectastain Universal Elite ABC kit, Vector Laboratories, Newark, CA, USA); the signal was visualised using 3,30-diaminobenzidine (DAB) (Vector Laboratories, Newark, CA, USA). A summary of the primary antibodies, all commercially available and optimised for this study, is shown in Table 4. Negative controls, including omission of the primary antibody and isotype controls, were performed.

Expression of neuronal p21 associated with neurofibrillary tangles and expression of p16 with markers of astrocytes (glial fibrillary acidic protein (GFAP)) and microglia (ionised calcium binding adaptor molecule 1 (Iba1)) were assessed by dual immune labelling. For this, p21 detection was performed on FFPE tissue as described previously using a standard avidin–biotin horseradish peroxidase enzyme complex method (Vectastain Universal Elite ABC kit, Vector Laboratories, Newark, CA, USA) and the signal was visualised with DAB (Vector Laboratories, Newark, CA, USA). Tissue sections were thoroughly rinsed with distilled water, incubated with the avidin–biotin blocking kit (Vector Laboratories, Newark, CA, USA) and blocked with normal 1.5% serum for 45 min at RT. This was followed by incubation with anti-AT8/GFAP/Iba1 antibodies respectively (Table 1) for 1 h at room temperature, and detected using a standard avidin–biotin alkaline phosphatase method with Vector Red Alkaline Phosphatase (AP) substrate detection system (Vector Laboratories, Newark, CA, USA).

### 4.3. Quantification

FFPE-stained sections were scanned with a NanoZoomer XR Digital slide scanner (Hamamatsu, Welwyn Garden City, UK) and visualised with the NDP.view2 Image viewing software version 2.10.0 (Hamamatsu, Welwyn Garden City, UK). For quantification of all markers, the scanned sections were loaded on to the Quantitative Pathology and Bioimage Analysis (QuPath) software version 0.6.0 [53] to detect and quantify positively stained glial and neuronal cells separately using size exclusion (cut-off 65 μm^2^), as used previously [36]. Results were reported as percentage of positive cells (glial or neuronal) per mm^2^ for each marker assessed.

### 4.4. Statistical Analysis

Statistical analyses were conducted using two software programs: IBM-SPSS (version 28, IBM, Portsmouth, UK) and R Studio version 2025.5.1.513.3 (Posit PBC, Boston, MA, USA). The normality of the data was assessed using the Kolmogorov–Smirnov test. Since most of the data did not follow a normal distribution, Spearman’s rank test was used for correlation analyses and the Kruskal–Wallis test was used to compare variation between groups. To account for multiple comparisons, *p*-values from post hoc testing were corrected using the Bonferroni method. The Wilcoxon signed-rank test was used to compare continuous data between two groups. In the analysis of senescence and DNA damage markers with Braak NFT stage, the six stages were combined into three groups: entorhinal stages (Braak stages 0–2; 30 cases), limbic stages (Braak stages 3–4; 51 cases), and isocortical stages (Braak stages 5–6; 18 cases). Similarly, for the analysis of the Thal phase, the phases were combined into three groups: no- or only-cortical Aβ (phases 0–1; 19 cases), hippocampal/limbic Aβ (phases 2–3; 33 cases), and midbrain/cerebellar involvement (phases 4–5; 27 cases). The relationships between the outcomes of senescence and DNA damage markers with dementia and neuropathology were investigated using logistic regression, controlling the analyses for age at death and sex. All tests were two-tailed, with a *p* value of <0.05 regarded as significant.

### 4.5. Assessing the Transcriptomic Profile Associated with Neuronal p21 Expression

Based on the p21 immunoreactive profile, transcriptomic analysis was performed on pyramidal and small neurons isolated from the contralateral snap frozen frontal cortex of 6 cases with high and 6 low levels of neuronal senescence using laser capture microdissection (LCM). Cases were selected to include those with the best RNA quality and tissue pH, and to include both males and females in both groups. The case characteristics, including brain pH and post-LCM ribosomal integrity number, are shown in Supplementary Table S6).

Neurons were isolated from toluidine blue-stained frozen sections (7 µm) using the Veritas laser-capture microdissection system (Arcturus Bioscience, Mountain View, CA, USA). Total RNA was extracted using the PicoPure RNA isolation kit, according to the manufacturer’s instructions (Arcturus BioScience, Mountain View, CA, USA), and the quantity and quality assessed using a Nanodrop 1000 spectrophotometer (Thermoscientific, Hemel Hempstead, UK) and a 2100 Bioanalyser, respectively (Agilent, Santa Clara, CA, USA). The target labelled mRNA was prepared following the GeneChip WT Pico amplification protocol (Affymetrix, Altrincham, UK) using low-cycle PCR followed by amplification using T7 in vitro transcription (IVT). The cRNA was converted to biotinylated sense-strand DNA hybridisation targets. Approximately 3–6 μg of DNA was amplified, fragmented, labelled and hybridised to Affymetrix Clariom D Array chips and the fluorescence intensity of hybridised transcripts assessed using the GeneChip 3000 scanner (Thermofisher Scientific, Hemel Hempstead, UK).

The .CEL files were imported into Transcription Analysis Console (TAC)version 4.0.1 (Applied Biosystems, Foster City, CA, USA) and normalised to the median of all genes, prior to statistical analysis. Transcripts were defined as significantly differentially expressed genes (DEGs) if they had a fold change ≥ ±1.2 and *p*-value ≤ 0.05. DEGs were inputted into the Database for Annotation Visualisation and Integrated Discovery (DAVID) using Kyto Encyclopaedia of Genes and Genomes (KEGG) pathway analysis to identify specific pathways and Functional Annotation Clustering (FAC) to identify functionally similar gene groups. In the case of a significant result, the *p* value was adjusted for multiple testing using the Bonferroni correction and considered significant if *p* ≤ 0.05.

## Figures and Tables

**Table 1:** Sample size (n), proportion of sample, median expression and inter-quartile range for glial senescence markers over Braak stage, Thal phase, CAA and dementia status *p*-values for Braak stage, Thal stage and CAA calculated by Kruskal–Wallis test; *p*-values for dementia status calculated by Wilcoxon rank-sum test. Proportions calculated over the total (n = 273) of available samples.

		Glial p21						Glial yH2AX					
		n	Proportion (%)	Median	p25	p75	*p* =	n	Proportion (%)	Median	**p25**	**p75**	***p*** **=**
Braak Stage	0–II	78	28.57	12.51	2.62	39.74	0.02	76	28.57	15.89	1.03	47.42	0.02
	III–IV	91	33.33	8.22	1.48	30.96		92	34.59	15.65	4.53	44.00	
	V–VI	48	17.58	4.01	1.11	10.95		47	17.67	10.79	3.05	22.90	
Thal Phase	0–I	32	11.72	8.77	1.25	28.27	0.49	32	12.03	14.91	0.85	58.74	0.93
	II–III	58	21.25	8.64	1.02	26.30		58	21.80	20.42	0.83	49.04	
	IV–V	63	23.08	5.57	1.19	20.31		63	23.68	13.95	2.22	43.29	
CAA	Low	52	19.05	9.97	2.11	39.36	0.23	52	19.55	19.59	0.65	58.74	0.99
	Moderate	59	21.61	7.61	0.81	20.53		59	22.18	18.36	1.45	47.66	
	High	40	14.65	6.81	1.28	14.17		40	15.04	12.04	3.10	43.28	
Dementia Status	No dementia	115	42.12	7.20	1.37	39.74	0.33	111	41.73	9.36	1.99	30.59	0.91
	Dementia	158	57.88	8.46	1.06	23.47		155	58.27	11.51	2.79	30.89	

**Table 2:** Sample size (n), proportion of sample, median expression and inter-quartile range for neuronal senescence markers over Braak stage, Thal phase, CAA and dementia status. *p*-values for Braak stage, Thal stage and CAA calculated by Kruskal–Wallis test, *p*-values for dementia status calculated by Wilcoxon rank-sum test. Proportions calculated over the total (n = 273) of available samples.

		Neuronal p21						Neuronal yH2AX					
		n	Proportion (%)	Median	p25	p75	*p* =	n	Proportion (%)	Median	**p25**	**p75**	***p*** **=**
Braak Stage	0–II	78	28.57	21.49	4.04	60.97	0.08	76	28.57	7.15	0.72	29.01	0.38
	III–IV	91	33.33	14.63	5.27	50.51		92	34.59	9.44	2.70	27.66	
	V–VI	48	17.58	10.51	2.51	26.20		47	17.67	6.45	2.09	17.53	
Thal Phase	0–I	32	11.72	10.62	4.80	47.42	0.58	32	12.03	4.70	0.62	44.29	0.90
	II–III	58	21.25	19.73	4.17	56.09		58	21.80	5.94	0.43	31.81	
	IV–V	63	23.08	12.26	4.59	35.41		63	23.68	5.13	1.37	24.52	
CAA	Low	52	19.05	13.50	5.00	62.77	0.22	52	19.55	7.33	0.33	34.74	0.97
	Moderate	59	21.61	8.80	2.18	36.36		59	22.18	4.71	0.59	24.52	
	High	40	14.65	14.52	5.30	50.68		40	15.04	5.05	1.30	27.12	
Dementia Status	No dementia	115	42.12	12.37	3.78	54.68	0.26	111	41.73	7.12	0.97	24.52	0.92
	Dementia	158	57.88	12.46	3.19	34.16		155	58.27	6.00	1.71	22.91	

**Table 3:** Logistic regression results for risk of dementia, univariate and multivariate, adjusted for Braak stage, Thal phase and CAA. Senescence markers were modelled on a continuous scale and as tertiles.

		Unadjusted		Adjusted	
Marker		OR	95% CI	OR	95% CI
Neuronal P21	Continuous	0.99	(0.98–1.00)	1.00	(0.99–1.01)
	Low	1.00	.	1.00	.
	Moderate	1.32	(0.73–2.41)	1.29	(0.50–3.35)
	High	0.73	(0.41–1.32)	1.14	(0.44–2.91)
Neuronal yH2AX	Continuous	1.00	(0.99–1.01)	1.01	(0.99–1.02)
	Low	1.00	.	1.00	.
	Moderate	1.77	(0.96–3.25)	2.58	(0.95–7.05)
	High	0.98	(0.54–1.77)	1.66	(0.69–3.98)
Glial P21	Continuous	0.99	(0.98–1.00)	1.00	(0.98–1.02)
	Low	1.00	.	1.00	.
	Moderate	1.26	(0.69–2.30)	1.85	(0.72–4.74)
	High	0.67	(0.37–1.20)	1.50	(0.57–3.95)
Glial yH2AX	Continuous	1.00	(0.99–1.01)	1.01	(0.99–1.02)
	Low	1.00	.	1.00	.
	Moderate	1.33	(0.73–2.43)	1.73	(0.60–4.98)
	High	0.85	(0.47–1.55)	1.87	(0.78–4.44)

**Table 4:** Antibodies and conditions used for immunohistochemical and immunofluorescence detection of senescence and DNA damage markers. TSC = tri-sodium citrate; RT= room temperature; NA = not applicable.

Antibody	Antigen Retrieval	Concentration	Incubation
Anti-p21 (Abcam ab109520, rabbit monoclonal)	TSC buffer pH6, pressure cooker	1:100	Overnight at 4 °C
Anti-p16 (Biogenex AM540-10M, mouse monoclonal)	Tris-EDTA buffer pH9, microwave 15 min	Pre-diluted	Overnight at 4 °C
Anti-H3K9me3 (Abcam ab8898, rabbit polyclonal)	TSC buffer pH6, microwave 20 min	1:500	Overnight at 4 °C
Anti-γH2AX (R&D Systems AF2288, rabbit polyclonal)	Tris-EDTA buffer pH8, pressure cooker	1:500	1 h, RT
Anti-GFAP (Dako Z0334, rabbit polyclonal)	NA (used for double labelling on FFPE tissue)	1:500	1 h, RT
Anti-Iba1 (Millipore MABN92, mouse monoclonal)	NA (used for double labelling on FFPE tissue)	1:100	1 h, RT
Anti-AT8 (Thermo-Fisher MN1020, mouse monoclonal)	NA (used for double labelling on FFPE tissue)	1:200	1 h, RT

## Data Availability

The data that support the findings of this study are available on request from the corresponding author. The data are not publicly available due to ethical restrictions of CFAS.

## Supplementary Materials

The following supporting information can be downloaded at: https://www.mdpi.com/article/10.3390/ijms27156964/s1.

## Abbreviations

The following abbreviations are used in this manuscript:

ADNC	Alzheimer’s disease neuropathological change
ARTAG	Aging-related tau astrogliopathy
CAA	Cerebral amyloid angiopathy
CFAS	Cognitive Function and Ageing Study
DEGs	Differentially expressed genes
FFPE	Formalin-fixed paraffin embedded
GFAP	Glial fibrillary acidic protein
Iba1	Ionised calcium binding adaptor protein 1
LATE	Limbic-predominant age-related TDP-43 encephalopathy
NFT	Neurofibrillary tangle
SAHF	Senescence-associated heterochromatin foci
SASP	Senescence-associated secretory phenotype
