# Supplementary material for: Senescence Markers and Associated Transcriptomic Changes Are Expressed at Early Stages of Alzheimer’s Neuropathology but Are Not Independently Related to Dementia
Source: Int J Mol Sci. 2026 Aug 3;27(15):6964. doi: 10.3390/ijms27156964 (PMC13466898; doi:10.3390/ijms27156964)
Supplement: Supplementary file 1 [file ijms-27-06964-s001.zip › ijms-4441618-supplementary.pdf]

Supplementary Figure S1. Distributions of senescence marker measures within the cohort

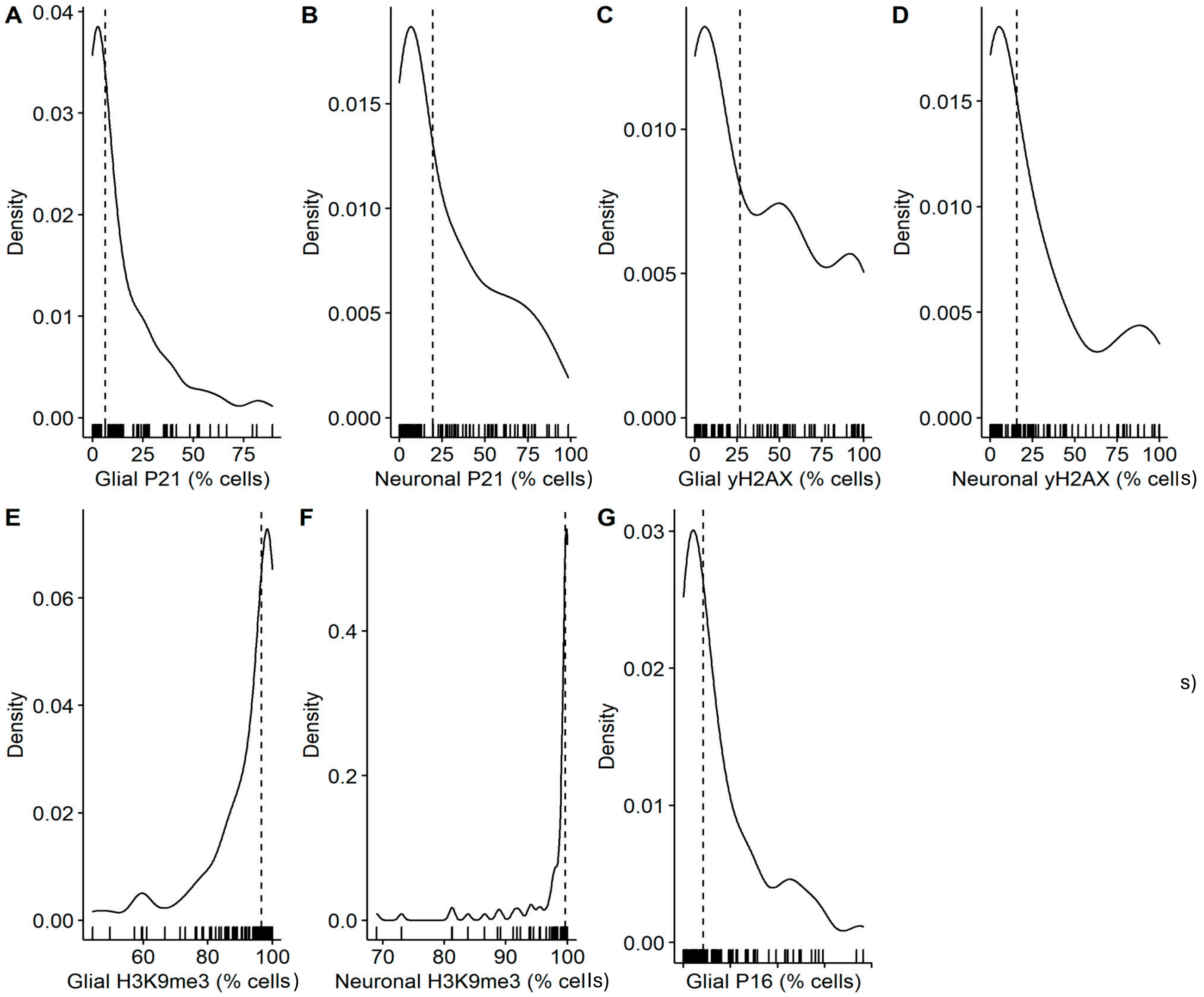

Supplementary Table S1: Sample size for senescence markers, median expression with interquartile range

Marker	n	Median	p25	p75
Neuronal P21	273	12.37	3.75	41.14
Neuronal yH2AX	266	6.46	1.53	23.92
Glial P21	273	8.05	1.19	29.51
Glial yH2AX	266	11.15	2.60	30.59

Supplementary Table S2. KEGG pathway analysis of DEGs in neurones (pyramidal and small neurones combined) associated with high measures of neuronal senescence

KEGG pathway	No. genes	p-value	Benjamini
Parkinson disease	113	$2.3x10^{-24}$	$8.2x10^{-22}$
Huntington disease	119	$7.1x10^{-22}$	$1.3x10^{-19}$
Pathways of neurodegeneration	157	$2.4x10^{-20}$	$2.8x10^{-18}$
Prion disease	106	$2.1x10^{-19}$	$1.9x10^{-17}$
Alzheimer disease	131	$2.9x10^{-18}$	$2.1x10^{-16}$
Amyotrophic lateral sclerosis	121	$9.5x10^{-16}$	$5.7x10^{-14}$
Oxidative phosphorylation	59	$1.7x10^{-13}$	$8.8x10^{-12}$
Non-alcoholic fatty liver disease	61	$8.9x10^{-12}$	$4.0x10^{-10}$
Protein processing in endoplasmic reticulum	64	$2.3x10^{-11}$	$9.2x10^{-10}$
Retrograde endocannabinoid signalling	57	$9.4x10^{-11}$	$3.4x10^{-9}$
Reactive oxygen species	75	$2.5x10^{-10}$	$8.2x10^{-9}$
Endocytosis	79	$1.3x10^{-9}$	$3.8x10^{-8}$
Diabetic cardiomyopathy	68	$1.5x10^{-9}$	$4.4x10^{-8}$
Calcium reabsorption	28	$3.3x10^{-9}$	$8.6x10^{-8}$
Thermogenesis	73	$7.4x10^{-9}$	$1.7x10^{-7}$
Ubiquitin mediated proteolysis	51	$1.2x10^{-8}$	$2.8x10^{-7}$
Metabolic pathways	327	$4.2x10^{-8}$	$8.8x10^{-7}$
Spinocerebellar ataxia	49	$1.7x10^{-7}$	$3.4x10^{-6}$
Synaptic vesicle cycle	32	$4.9x10^{-7}$	$9.2x10^{-6}$
Salmonella infection	71	$8.3x10^{-7}$	$1.5x10^{-5}$
Autophagy - animal	51	$5.1x10^{-6}$	$8.8x10^{-5}$
Cardiac muscle contraction	32	$5.4x10^{-6}$	$8.9x10^{-5}$
Proteasome	21	$8.4x10^{-6}$	$1.3x10^{-4}$
IgSF CAM signaling	74	$1.1x10^{-4}$	$1.7x10^{-3}$
Protein export	15	$1.2x10^{-4}$	$1.7x10^{-3}$
Oxytocin signaling pathway	44	$1.2x10^{-4}$	$1.7x10^{-3}$
Dopaminergic synapse	39	$1.5x10^{-4}$	$2.0x10^{-3}$
Vibrio cholerae infection	20	$1.8x10^{-4}$	$2.3x10^{-3}$
Mitophagy - animal	32	$3.2x10^{-4}$	$4.0x10^{-3}$
Long-term potentiation	23	$4.7x10^{-4}$	$5.6x10^{-3}$
N-Glycan biosynthesis	20	$7.1x10^{-4}$	$8.2x10^{-3}$
Shigellosis	61	$7.4x10^{-4}$	$8.3x10^{-3}$
Adrenergic signaling in cardiomyocytes	41	$8.9x10^{-4}$	$9.6x10^{-3}$
Tight junction	44	$1.0x10^{-3}$	$1.1x10^{-2}$
GABAergic synapse	27	$1.1x10^{-3}$	$1.2x10^{-2}$
Nucleotide excision repair	21	$1.3x10^{-3}$	$1.3x10^{-2}$
Pyruvate metabolism	17	$1.8x10^{-3}$	$1.7x10^{-2}$
Glutamatergic synapse	32	$2.0x10^{-3}$	$1.9x10^{-2}$
Nucleocytoplasmic transport	30	$2.5x10^{-3}$	$2.3x10^{-2}$
Pathogenic Escherichia coli infection	49	$2.5x10^{-3}$	$2.3x10^{-2}$
Thyroid hormone synthesis	22	$5.6x10^{-3}$	$4.9x10^{-2}$

Supplementary Table S3. Functional annotation of DEGs (pyramidal and small neurones combined) associated with high measures of senescence

Biological process	No. genes	p-value	Benjamini
Transport	482	$1.6x10^{-16}$	$2.8x10^{-14}$
Host-virus interaction	193	$9.9x10^{-15}$	$8.7x10^{-13}$
Respiratory chain	40	$3.2x10^{-14}$	$1.9x10^{-12}$
Protein transport	179	$1.1x10^{-12}$	$4.6x10^{-11}$
Electron transport	51	$3.6x10^{-12}$	$1.3x10^{-10}$
Ubl conjugation pathway	180	$3.7x10^{-6}$	$1.1x10^{-4}$
ER-Golgi transport	36	$9.2x10^{-6}$	$2.3x10^{-4}$
DNA replication	32	$7.1x10^{-5}$	$1.5x10^{-3}$
Autophagy	49	$1.0x10^{-4}$	$2.0x10^{-3}$
mRNA processing	95	$2.3x10^{-4}$	$4.0x10^{-3}$
mRNA splicing	75	$8.1x10^{-4}$	$1.3x10^{-2}$
Protein biosynthesis	42	$9.3x10^{-4}$	$1.4x10^{-2}$
Zinc transport	11	$2.9x10^{-3}$	$3.7x10^{-2}$
Endocytosis	35	$3.0x10^{-3}$	$3.7x10^{-2}$

Supplementary Table S4. KEGG pathway analysis of all DEGs in pyramidal neurones associated with senescence

KEGG pathway	No. genes	p-value	Benjamini
Parkinson disease	56	$1.1x10^{-9}$	$4.0x10^{-7}$
Huntington disease	56	$1.7x10^{-7}$	3.010^{-5}
Non-alcoholic fatty liver disease	34	$1.1x10^{-6}$	$1.3x10^{-4}$
Oxidative phosphorylation	31	$1.7x10^{-6}$	$1.5x10^{-4}$
Pathways of neurodegeneration	72	$4.5x10^{-6}$	$3.1x10^{-4}$
Alzheimer disease	61	$6.6x10^{-6}$	$3.6x10^{-4}$
Protein processing in endoplasmic reticulum	34	$7.1x10^{-6}$	$3.6x10^{-4}$
Amyotrophic lateral sclerosis	58	$1.1x10^{-5}$	$4.7x10^{-4}$
Ubiquitin mediated proteolysis	29	$2.6x10^{-5}$	$1.0x10^{-3}$
Prion disease	45	$5.4x10^{-5}$	$1.9x10^{-3}$
Thermogenesis	39	$1.0x10^{-4}$	$3.2x10^{-3}$
Reactive oxygen species	38	$1.1x10^{-4}$	$3.3x10^{-3}$
Metabolic pathways	174	$1.9x10^{-4}$	$5.0x10^{-3}$

Supplementary Table S5: Functional annotation of DEGs in pyramidal neurones associated with senescence

Biological process	No. genes	p-value	Benjamini
Host-virus interaction	99	$3.0x10^{-7}$	$4.9x10^{-5}$
Respiratory chain	21	$6.9x10^{-7}$	$5.5x10^{-5}$
Electron transport	27	$2.6x10^{-6}$	$1.4x10^{-4}$
Transport	228	$6.4x10^{-5}$	$2.6x10^{-3}$
Protein transport	82	$3.2x10^{-4}$	$1.0x10^{-2}$

Supplementary Table S6. Cases for transcriptomic analysis

Neuronal Senescence	Age (y)	Sex (M/F)	pH	Post-LCM RIN
**Low p21 cases**				
1	71	M	6.54	2.5
2	93	M	6.6	2.6
3	77	F	6.34	2.5
4	75	M	6.2	2.4
5	91	M	6.23	2.5
6	88	F	6.14	2.6
**High p21 cases**				
1	82	F	6.45	2.6
2	76	M	6.92	2.6
3	71	F	6.49	2.5
4	87	F	6.7	2.6
5	88	F	6.1	2.3
6	80	M	6.6	2.5
